# A Systematised Review of Primary School Whole Class Child Obesity Interventions: Effectiveness, Characteristics, and Strategies

**DOI:** 10.1155/2016/4902714

**Published:** 2016-09-07

**Authors:** Elise C. Brown, Duncan S. Buchan, Julien S. Baker, Frank B. Wyatt, Danilo S. Bocalini, Lon Kilgore

**Affiliations:** ^1^Department of Wellness, Health Promotion and Injury Prevention, School of Health Sciences, Oakland University, 2200 N. Squirrel Road, Rochester, MI 48309-4452, USA; ^2^Institute for Clinical Exercise & Health Science, University of the West of Scotland, Almada Street, Hamilton ML3 0JB, UK; ^3^Department of Exercise Physiology & Athletic Training, Midwestern State University, Wichita Falls, TX 76308, USA; ^4^Translational Physiology Laboratory and Post Graduate Program in Physical Education and Aging Science, São Judas Tadeu University (USJT), 05503-001 São Paulo, SP, Brazil; ^5^Kilgore Academy, 816 Keli Court, Azle, TX 76020, USA

## Abstract

*Background*. A systematised review was conducted to examine the effectiveness of school-based interventions that focus on changing dietary intake and physical activity levels to reduce childhood obesity.* Methods*. Multiple databases were searched for randomised and nonrandomised interventions from 2007 to 2016 in full-time elementary schools, which were delivered to the whole class, included dietary and physical activity components, involved both sexes, were written in English, and used body mass index (BMI) as an outcome.* Results*. The database search produced 8,866 titles from which 78 were deemed relevant and assessed for inclusion resulting in 15 studies meeting all inclusion criteria. From these 15 studies, 9 yielded a reduction or stabilisation in BMI or BMI *z*-score in the entire intervention group and/or subgroups. Programmes lasting between 6 and 12 months that involve multiple environmental, educational, and physical strategies appear to be most likely to result in BMI or BMI *z*-score improvement. Moderators most likely influencing an improvement in BMI included increased physical activity, decreased sugar sweetened beverages intake, and increased fruit intake.* Conclusions*. School-based interventions may be an effective means for child obesity prevention. The identification of consistent elements used in school-based interventions that have demonstrated effectiveness may aid in preventing child obesity.

## 1. Introduction

From 1980 to 2013, child obesity prevalence increased by 47.1% globally [1]. The elevated prevalence rates are concerning because of the associated increased risk of obese children developing dyslipidemia, hypertension, and insulin resistance compared to normal weight children [2]. Additionally, when child obesity continues into adulthood the individual is at greater risk of health complications [3]. When estimating incidence of child obesity, children are often classified as obese based on body mass index (BMI) percentile cut-offs from growth references [4–7]. BMI and BMI standard deviation scores (BMI-SDS) or *z*-scores are primary methods for governments [7, 8] and international health organisations [5, 6] to classify children as obese. BMI is also the most commonly used obesity indicator for clinical and research purposes [9].

Although it has been recognized that schools are ideal settings for obesity prevention initiatives [10], systematic reviews and meta-analyses have demonstrated mixed results in terms of the effectiveness of school-based child obesity treatment and prevention interventions [11–14]. Hung and colleagues concluded that school-based interventions have not been effective in improving BMI [13]. On the other hand, Lavelle and colleagues reported that school-based interventions are effective in reducing BMI [14], and Gonzalez-Suarez concluded that school-based interventions are effective in the short-term at obesity prevention [12]. However, each of these studies included universal interventions (delivered to the whole class) as well as interventions delivered specifically to obese children. Interventions including only obese children are likely to bias components towards treatment rather than combining prevention and treatment strategies typical to universal approaches [14], and these types of interventions may need to be assessed separately. Brown and Summerbell only included universal studies and concluded that physical activity (PA) interventions may be an effective means for overweight prevention [11]. Although this review focused solely on universal interventions, only interventions up to the year 2007 were included in the study. Therefore, an updated review including only universal interventions is needed. The primary aim of this review is to assess the effects of universal, school-based interventions with healthy eating (HE) and PA components for the prevention and treatment of obesity in primary school children. A secondary aim is to identify intervention characteristics and moderators that may contribute to effectiveness.

## 2. Materials and Methods

The inclusion criteria for systematic reviews such as Cochrane reviews are driven by the participants, interventions, and clinical questions being asked [15]. The use of another method of qualitative reviews, systematised reviews, was chosen for this focused approach requiring the outcome measure to be part of the inclusion criteria. In order to maintain quality and reduce bias [16], all processes were described in detail and quality assessment of included studies was conducted. PRISMA guidelines were used for the reporting procedures [17].

### 2.1. Literature Search

A literature search was conducted in PubMed, Health Source, MEDLINE, PsycBOOKS, Psychology and Behavioural Sciences Collection, PsycINFO, SocINDEX, and SPORTDiscus in the years 2007–2016. Various search terms were used including “child overweight,” “child obesity,” “physical activity,” “nutrition,” “health education,” “BMI,” “BMI *z*-score,” “BMI-SDS,” and “school intervention,” and the search was limited to peer-reviewed journal articles. Also, reviews and meta-analyses were cross-referenced to identify additional studies that were not previously captured.

### 2.2. Inclusion Criteria

Given the widespread use and the impact that BMI may have on government policy [18], a focused approach was taken which limited the outcome variables to BMI and BMI-SDS/*z*-score. From this point forward, BMI and BMI-SDS/*z*-score will be referred to as BMI unless BMI-SDS/*z*-score is specified. Studies selected for inclusion were school-based, universal initiatives which aimed to improve BMI and included PA and HE components. Studies must have included BMI in pre- and postanalyses. Articles examining changes in obesity prevalence without providing BMI changes were not included. Since participants who attend after-school lifestyle programmes may have different characteristics compared to those who do not, such as higher PA levels [19], exclusively after-school studies were excluded. Interventions must have had HE and PA component during school hours. Studies that met these criteria but additionally had after-school PA programmes were also included. Participants included boys and girls of any nationality in full-time elementary schools. Studies must have been written in English. Study designs included randomised controlled trials (RCTs) and nonrandomised controlled trials (NRCTs) with no-intervention controls. Studies including multiple treatment groups without a control group were excluded.

### 2.3. Intervention Characteristics

Intervention duration was classified as short-term (≤6 months), moderate-term (>6 months and ≤12 months), or long-term (>12 months) [20]. Studies were classified as teacher-led if the intervention was delivered by classroom or physical education teachers or if the teacher collaborated with other professionals or students [21]. If no parental involvement was described, the study was classified as no parental involvement [13]. Studies were classified as no theoretical framework if there was no mention of the use of a behaviour change theory or theoretical framework [22]. Intervention types were classified as educational intervention, environmental intervention, physical intervention, or a combination of the three [14].

### 2.4. Outcome Measures

Primary outcomes investigated to determine intervention success included BMI and/or BMI-SDS/*z*-score. In line with Demetriou and Höner's review, intervention success was defined as a reduction in BMI for the intervention group when compared with the control group or no change in BMI for the intervention group when compared to an increase in the control group [23]. Other outcomes such as no significant changes in BMI for the intervention and control group were labelled as no change.

### 2.5. Moderator Variables

Behavioural moderators included PA, fruit intake, vegetable intake, sedentary time, screen time (including TV viewing time only), and sugar sweetened beverage (SSB) intake. Multiple articles relating to the same study were included if relative outcomes were published separately.

### 2.6. Study Quality

To determine the validity and quality of individual studies, Downs and Black's validated tool for assessing the methodological quality of randomised and nonrandomised studies of health care interventions was used [24]. Subscales of the tool examined reporting, external validity, internal validity bias, selection bias, and power. Item 27 of the tool assessed power and had 6 possible scores based on the sample size. In line with Marquet and colleagues, scoring for item 27 was simplified so that a score of one was given if sufficient statistical power was achieved and a score of zero was given if sufficient power was not achieved [25]. The tool included a total of 27 items, and items were scored one or zero with a higher score indicative of higher quality. See the following list for criteria used. In accordance with HaiBo and colleagues, if studies received a score of one on at least 50% of the items then they were deemed sufficient in quality and were included in the review [26].

 Criteria for assessing study quality and bias are as follows:

 Reporting: it included the following points:

1. Is the hypothesis/aim/objective of the study clearly described?
2. Are the main outcomes to be measured clearly described in the introduction or methods section?
3. Are the characteristics of the schools/students included in the study clearly described?
4. Are the interventions of interest clearly described?
5. Are the distributions of principal confounders in each group of subjects to be compared clearly described?
6. Are the main findings of the study clearly described?
7. Does the study provide estimates of the random variability in the data for the main outcomes?
8. Have all important adverse events that may be a consequence of the intervention been reported?
9. Have the characteristics of patients lost to follow-up been described?
10. Have actual probability values been reported for main outcomes except where the probability value is <0.001?

External validity: it included the following points:

(11) Were the subjects asked to participate in the study representative of the entire population from which they were recruited?
(12) Were those subjects who were prepared to participate representative of the entire population from which they were recruited?
(13) Were the staff, places, and facilities, where the patients were treated, representative of the treatment the majority of patients receive?

Internal validity-bias included the following points:

(14) Was an attempt made to blind study subjects to the intervention they have received?
(15) Was an attempt made to blind those measuring the main outcomes of the intervention?
(16) If any of the results of the study were based on “data dredging,” was this made clear?
(17) In trials and cohort studies, do the analyses adjust for different lengths of follow-up of patients, or, in case-control studies, is the time period between the intervention and outcome the same for cases and controls?
(18) Were the statistical tests used to assess the main outcomes appropriate?
(19) Was compliance with the interventions reliable?
(20) Were the main outcome measures used accurate (valid and reliable)?

Internal validity-confounding (selection bias) included the following points:

(21) Were the patients in different intervention groups (trials and cohort studies) or were the cases and controls (case-control studies) recruited from the same population?
(22) Were study subjects in different intervention groups (trials and cohort studies) or were the cases and controls (case-control studies) recruited over the same period of time?
(23) Were study subjects randomised to intervention groups?
(24) Was the randomised intervention assignment concealed from both patients and health care staff until recruitment was complete and irrevocable?
(25) Was there adequate adjustment for confounding in the analyses from which the main findings were drawn?
(26) Were losses of patients to follow-up taken into account?

Power: it included the following:

(27) Did the study have sufficient power to detect a clinically important effect where the probability value for a difference being due to chance is less than 5%?

### 2.7. Analysis Plan

A qualitative analysis of the findings was conducted. Similar to Golley and colleagues approach [27], a behavioural variable was classified as a potential moderator if a significant change in the variable occurred in addition to a significant change in BMI in the intervention compared to the control group. The frequency of intervention effectiveness by moderator variable was determined. Data was extracted by one reviewer and summarized from each article. The extracted data included intervention length, delivery personnel, theoretical framework, study design, strategies, components, and outcomes. The results were presented in narrative form.

## 3. Results

**Figure 1:**
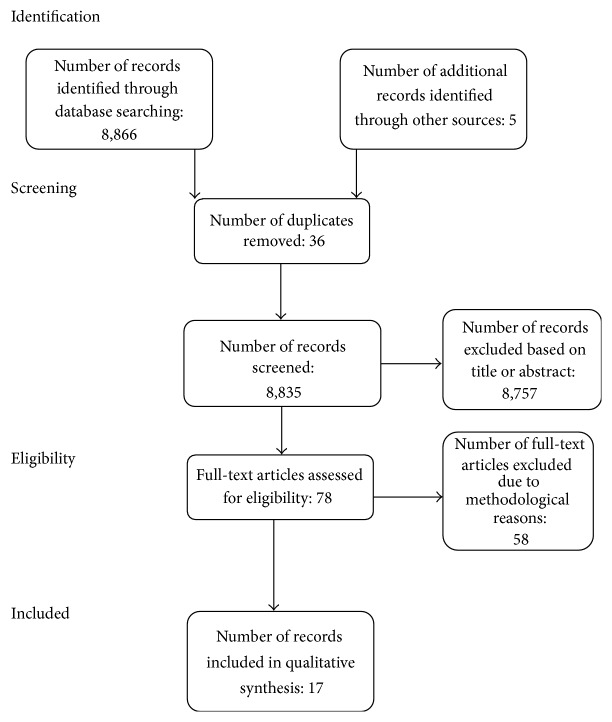
PRISMA flow diagram of processes for study inclusion.

See Figure 1 for a description of study selection and processing. Fifteen studies met the inclusion criteria and were synthesized in this review. Additionally, 2 other studies [28, 29] were included as they related to one specific intervention [30] that met all inclusion criteria and elaborated on moderators. In total, 17 studies were included.

### 3.1. Intervention Characteristics

Table 1 provides intervention methodologies, characteristics, strategies, and critical appraisal scores in alphabetical order of study location. From the included studies the sample sizes ranged from 294 to 2622 participants, and the intervention durations ranged from 5 to 36 months. Seven studies had intervention durations >12 months with five resulting in improved BMI [30–34], seven studies lasted between 6 and 12 months with four achieving intervention effectiveness [35–38], and one study's duration was < six months with no BMI improvement [39]. Eight studies utilized a behaviour change theory [30, 33, 35, 36, 39–42] with the Social Cognitive Theory (SCT) being the most frequently used. Four of these studies resulted in an improvement in BMI [30, 33, 35, 36]. Similarly, 5 of the 9 studies that did not include a behaviour change theory resulted in BMI improvement [31, 32, 34, 37, 38]. Most interventions were delivered solely by teachers, while some were delivered by teachers and an internet programme [34], teachers alongside exercise and nutrition specialists [43], and teachers and older students [38]. Seven of the twelve studies that included teachers-led interventions were effective [30, 33–38]. Non-teacher delivered interventions were delivered by community activity coordinators and undergraduate medical students, and one study only made environmental changes. Eleven studies included a parental involvement component. Although six of the eleven interventions including a parental component prevented a decrease in or improved BMI [30, 31, 33, 35–37], three out of the four studies that did not include parental involvement also noted improvements in BMI [32, 34, 38]. Most studies used a combination of environmental, educational, physical activity, and parental involvement strategies, while four used only an educational strategy. Eight of the eleven studies that used a combination of strategies achieved effectiveness [30, 32–38], and one of the four that used only educational strategies was effective [31]. All studies met the criteria for methodological quality.

### 3.2. Primary Outcomes

Table 2 presents the primary and secondary variables and outcomes. Some studies reported BMI changes for subgroupings rather than the entire group. When looking at BMI in the total intervention group, six out of fourteen studies achieved an improvement [31–33, 35–37]. Nine out of fifteen studies resulted in an improvement in BMI in the total group or subgroupings [30–38]. In studies that analysed subgroupings, some reported that improvements in BMI were greater for older children [38], girls [30], and white girls [34], while others found neither sex differences [37] nor weight status differences [34].

### 3.3. Moderator Variables

Ten studies measured total PA or moderate and vigorous PA (MVPA), and 4 of these studies resulted in improvements in these variables alongside improvement in BMI in the whole intervention group [33, 35–37]. Two of the four studies measuring SSB intake reported improved BMI alongside decreased SSB intake [32, 36]. Eight studies measured fruit and vegetable intake, and three achieved an increase in fruit intake alongside BMI improvement [32, 33, 36], while no studies resulted in increased vegetable intake alongside BMI improvement. None of the three studies measuring sedentary behaviour [34, 39, 40] nor the 1 measuring total screen time [32] achieved a decrease in any of these variables. One study that measured weekend and weekday screen time separately achieved a decrease in weekend screen time, no change in weekday screen time, and no change in BMI [40].

### 3.4. Sustained Intervention Effects

One study reported follow-up measures once the intervention ceased [39]. CHANGE! lasted 5 months and did not achieve a reduction in BMI or BMI *z*-score after measures but reported a significantly lower BMI *z*-score at 10 weeks after intervention.

## 4. Discussion

The findings of the current systematised review suggest that school-based interventions that include HE and PA components are moderately effective methods for improving BMI in elementary school children which is consistent with the findings of others [11, 14]. Similarly, Brown and Summerbell's review suggested that school-based obesity interventions containing HE and PA components may help prevent overweight [11], and Lavelle et al. also determined that school-based interventions may be effective in reducing BMI [14].

Only one study reported age differences [38] and, therefore, no conclusions could be made in terms of effectiveness in different age groups. Two studies found that intervention effectiveness was greater in girls [30, 34] which aligned with another review [11], while one study reported no sex differences [37]. Grydeland et al.'s [30] and Williamson et al.'s [34] findings were consistent with Brown and Summerbell's [11] conclusions of obesity interventions being more effective in girls. These findings may be due to the diverse nature of intervention approaches as Grydeland et al. [30] noted in the Health in Adolescents (HEIA) programme that the developers, implementers, and teachers involved were primarily women which may have unintentionally biased components towards girls. Gender bias has also been suggested by Befort who noted that since the 1980s adult obesity interventions may have been unintentionally favoured towards women [44]. Although limited evidence is available which suggests this is apparent in youth interventions, it is recommended that future work examine the potential for gender bias.

Moderators for BMI improvement included increased PA, lowered SSB intake, and increased fruit intake. The studies in this review that measured sedentary behaviour and screen time did not result in reductions in these behaviours or improvements in BMI. This is in contrast to DeMattia and colleagues' review that found that two of the three included elementary school studies were effective in reducing sedentary behaviours with one noting improvements in BMI [45]. The studies that were effective in reducing sedentary behaviours in the previously mentioned review intensively implemented techniques specific to reducing sedentary behaviour. It may be that school-based interventions with broader aims at improving multiple behaviours may not be intensive enough to reduce sedentary time. Nonetheless, further work is needed in order to identify ways to improve these variables.

PA and/or MVPA was the most reported moderator with six studies using objective measures (accelerometer or pedometer) and five studies using questionnaires. In those studies that captured objectively measured PA, three studies demonstrated improvements in PA alongside improvements in BMI. Reduction of SSB intake has been reported by parents and children to be one of the easiest health behaviours to modify [46] and it is encouraging that two out of four studies that measured SSB intake reported improvements in BMI alongside a decrease in SSB intake. A systematic review determined a positive association between SSB intake and obesity in children [47]. The link may be due to the high sugar content and low satiety associated with SSBs. Lowering SSB intake may be achievable through school-based initiatives and may help improve BMI.

None of the studies measuring vegetable intake increased this variable alongside BMI improvement. Increasing vegetable consumption appeared more difficult than increasing fruit intake which may be attributed to the child's perception of fruit being more palatable than vegetables [48].

Teachers play a strong role in a child's social environment and have the potential to positively influence behaviours through environmental and social interactions [49]. Teacher-led interventions were effective in improving BMI. They were also the most common delivery method and may be the most sustainable approach for long-term impact.

Multiple reviews have stressed the importance of basing child obesity interventions on behaviour change theories [27, 50, 51]. However, the improvement of BMI in this study did not seem to be impacted by the use of any theoretical approach. It may be that some researchers in this study used strategies based on behaviour change theories but failed to report the theoretical framework. The popular use of SCT was consistent with school-based interventions from 1999 to 2004 [50].

It was unclear how parental involvement influenced intervention effectiveness given the disparity between levels of parental involvement across studies. Six of the eleven studies that included a parental involvement component resulted in BMI improvement; however, three of the four studies that did not include a parental involvement component within their study design also reported improvements in BMI. These findings are in line with Cook-Cottone and colleagues review which found that interventions with minimal or moderate degrees of parental involvement achieved similar BMI results to those without a parental involvement component [21]. Additionally, other reviews have found that the degree of intervention effectiveness is related to the extent of parental involvement implemented [52, 53]. Although minimal parental involvement may be viewed as a more sustainable teacher-led method, more intensive efforts could increase intervention effectiveness.

While multiple combinations of environmental, educational, and physical strategies demonstrated the capacity to improve child BMI, education-only interventions may not be sufficient to induce behaviour change. In line with SCT, our findings suggest that if a child's environment does not support and reinforce new knowledge and attitudes from education and/or the child does not practice the new PA knowledge through performing PA in a supportive environment, the likelihood of inducing behaviour change may be low [54]. Long-term interventions that seek to increase PA and improve HE through behaviours such as decreasing SSB intake and increasing fruit intake through a combination of environmental, educational, and physical strategies may be effective in improving BMI.

A number of limitations must be considered. Methodological limitations included the absence of quantitative assessment, use of one reviewer, and the use of BMI as an obesity marker. Although a qualitative review by one reviewer limited the type of conclusions that could be drawn, this review's focused approach allowed for a detailed synthesis of the most widely used obesity indicator in school-based interventions. Additionally, the use of other obesity measures such as waist circumference, body fat %, or waist-to-height ratio may give a better representation of child disease risk [55].

Methodological limitations of studies included a lack of reporting of adverse events, reporting characteristics of participants lost to follow-up, blinding subjects and assessors to conditions, measuring intervention fidelity, concealing intervention assignment from schools, reporting if participants who agreed to take part were representative of the population, and taking participants lost to follow-up into account. Thorough reporting procedures and the control for biases that threaten internal validity will allow the reader to make a fair judgment of study findings. Although great effort is required to carry out high-quality studies in school-based interventions, it is possible that publication bias in terms of researchers not reporting negative findings in studies may have influenced the results of this review.

## 5. Conclusions

Findings from this systematised review suggest that long-term initiatives that include a parental component and involve multiple environmental, educational, and physical strategies may be the most promising for improving indices of adiposity in elementary school aged children. Future school-based interventions designed to improve children's weight status should focus efforts to increase PA, decrease sedentary behaviours, lower SSB intake, and increase fruit intake, as well as BMI improvement. Targeted moderators could include increasing PA, lowering SSB intake, and increasing fruit intake. Although it is unlikely that one specific school-based intervention can be effective across different cultures, the identification of these moderators that have demonstrated promise should be incorporated into future efforts in combating the perpetuation of child obesity.

## Figures and Tables

**Table 1:** Summary of study methodology, main findings, and critical appraisal scores.

Location	Study ID (author et al., year, intervention name, and citation number)	Participants (*n*)	Intervention length (months)	Theory	Primarily delivered by	Study design	Strategies	Intervention characteristics	Critical appraisal score (out of 27)
Canada, British Columbia	Stock et al., 2007, Healthy Buddies [38]	360	10	N/A	Teacher and older students	NRCT	ED, P	Educational intervention: older students received 45-minute healthy living lesson from the leader weekly; older students paired with younger students and taught the lesson to the younger students in 30-minute sessions, 21 lessons taught, school-wide healthy living theme day. Physical intervention: pairs participated in 30-min aerobic circuits twice a week and were encouraged to engage vigorously	19

Chile, Ñuñoa	Kain et al., 2014 [37]	1471	12	N/A	Teachers	RCT	ED, P, and PI	Educational intervention: 8 lessons of HE education for students lasting 90 minutes each. Physical intervention: PE teachers were trained to increased time in PE as well as increased MVPA during PE. Parental involvement: motivational, instructional meetings with parents totaling 45 minutes	21

England, Wigan Borough	Fairclough et al., 2013, CHANGE! [39]	318	5	Social Cognitive Theory	Teachers	RCT	ED, PI	Educational intervention: teacher training; weekly lessons lasting 60 minutes of healthy lifestyle curriculum with HE and PA topics. Parental involvement: homework assignments involving parents	22

England, northeast	Gorely et al., 2009 GreatFun2Run [35]	589	10	Social Cognitive Theory	Teachers	NRCT	EN, ED, P, and PI	Environmental intervention: local media campaign, running event promotion. Educational intervention: HE and PA components across the curriculum, interactive website. Physical intervention: PE centered around running. Parental involvement: an interactive website was available to parents highlighting the importance of HE and PA, child homework assignments to be completed with parents, information and child PA planner provided	19

England, southwest	Kipping et al., 2014, Active for Life Year 5 (AFLY5) [40]	2123	6	Social Cognitive Theory	Teachers	RCT	ED, PI	Educational intervention: 16 lesson plans for PA and HE were delivered over the course of 2-3 school terms. Information on HE and PA was provided to schools intended for newsletters. Parental involvement: interactive homework assignments were given intended to be completed with parents and other family members; information on HE and PA was provided to schools intended for parents	22

Greece, Ioannina	Angelopoulos et al., 2009, CHILDREN Study [36]	646	12	Theory of Planned Behaviour	Teachers	RCT	EN, ED, P, and PI	Environmental intervention: access to playgrounds. Fruits and fresh fruit juice were available in the school canteen. Educational intervention: cross-curricular approach across science, environmental, and PE curriculums; 1-2 hours each week and covered HE and PA topics. Approaches utilized to facilitate behaviour change are discussion, active learning, cues, modelling, guided practice, enactment, problem solving, goal setting, self-reevaluation, environmental reevaluation, arguments, direct experience, and mobilizing social support. Physical intervention: two 45-minute activity sessions during PE each week at a moderate intensity with a focus on fun. Parental involvement: take home assignments to be completed with guardians; parents were encouraged to make fruit and fresh fruit juice available at home; school events involving the family were also held as tasting sessions of different fruits and vegetables	19

Netherlands, Rotterdam	Jansen et al., 2011, Lekker fit [42]	2622	8	Theory of Planned Behaviour; Ecological Model of Egger and Swinburn	Teachers/PE teachers	RCT	ED, P, and PI	Educational intervention: health education curriculum including 3 lessons focused on HE, active living, and healthy lifestyle choices. Physical intervention: 3 PE lessons a week taught by a PE teacher; additional activities and sports coordinated with local sports clubs were offered outside school hours on a volunteer basis. Parental involvement: a fitness report card was sent home to parents including child's weight status, as well as annual health promotion events with local sports clubs	18

New Zealand, Otago	Taylor et al., 2007, APPLE [32]	730	24	N/A	Community activity coordinators	NRCT	EN, P	Environmental intervention: installed water coolers; new sport and games equipment being supplied; and discouraged SSBs and encouraged intake of fruits and vegetables. Physical intervention: encouraged PA at lunch, recess, and after school through introduction of new games, sports, and activities. Resources were given to teachers to incorporate activity in class	19

New Zealand, Waikato	Rush et al., 2012, Project Energize [43]	1352	23	N/A	Teachers, supported by exercise and nutrition specialists	RCT	EN, ED, P, and PI	Environmental intervention: specialists promoted active transport, active lunch, and peer leadership of PA outside of school; modifications to the canteen were made to provide healthier snacks; healthy fundraising options were also provided to teachers. Educational intervention: children received classroom lessons on HE during the same 3 weeks their parents attended nutrition sessions. Physical intervention: specialists supported teachers by modelling fundamental movement skills, ball games, fitness activities, and sport games and emphasized keeping all children moving throughout the sessions; teachers were also supported on how to manage children during activity sessions. Parental involvement: 3 information sessions were delivered to parents which included a practical nutrition session	19

Norway, southeast	Grydeland et al., 2013, Grydeland et al., 2014, and Bergh et al., 2012, HEIA^*∗*^ [28–30]	1528	20	Social Ecological Theory	Teachers	RCT	EN, ED, P, and PI	Environmental intervention: active commute to school campaign, new sporting equipment. Educational intervention: classroom lessons about PA and dietary behaviours once a month; classroom posters; and computer programme for 7th graders regarding healthy behaviours. Physical intervention: weekly classroom PA breaks for 10 minutes, training of teachers for PE. Parental involvement: parent information sheet	23

Portugal	Rosário et al., 2012 [41]	294	6	Health Promotion Model; Social Cognitive Theory	Teachers	RCT	ED	Educational intervention: nutrition intervention with a PA education component; teachers trained by researchers (36 hours) on intervention delivery and delivery to students (36 hours). 12 lessons included topics such as HE for children, drinking water, fruit and vegetables, foods with low nutritional quality, PA, screen time, and cooking healthy meals	21

Spain, Reus, Cambrils, Salou, and Vila-seca	Tarro et al., 2014, Education in Alimentation (EdAl) [31]	1939	36	N/A	Undergraduate medical students	RCT	ED, PI	Educational intervention: 8 healthy lifestyle topics—advancing healthy lifestyles, drinking healthy drinks and avoiding SSBs, improving vegetable and legume intake, eating more fruits and nuts and decreasing high sugar/high fat snacks, promoting healthy habits (PA, home meals), and increasing fruit, dairy, and fish consumption; lessons were delivered in four 1-hour education and activity sessions each year over the course of 3 years for a total of 12 sessions and were not a part of the curriculum; corresponding booklets were used by teachers throughout the year. Parental involvement: similar activities children participated in that were included in the educational booklet children participated in were intended for parents also	21

Spain, Granollers	Llargues et al., 2011, Avall [33]	509	24	Investigation, Vision, Action, and Change (IVAC) methodology	Teachers	RCT	EN, ED, and PI	Environmental intervention: sport/games equipment provided to school. Parental involvement: healthy recipes were given to families on a monthly basis as well as literature about local facilities and paths for physical activity	19

United States, Louisiana	Williamson et al., 2012, LA Health Project [34]	2060	28	N/A	Teachers + internet programme	RCT	EN, ED	Environmental intervention: modifications were made to cafeteria food and vending machines to increase fruit and vegetables and decrease fat; the PA environmental component included changes to PE curriculum with aims of increasing MVPA to at least 60 minutes/day and decreasing screen time to <2 hours each day. Educational intervention: the environment + education group received an internet based education programme and classroom instruction during class time	21

United States, South Dakota	Story et al., 2012, Bright Start [56]	454	24	N/A	Only environmental	RCT	EN, P, and PI	Environmental intervention: modifications were made to cafeteria offerings of low-fat foods, low-fat nonflavoured milk, serve recommended portion sizes, increase fruit and vegetable availability and only gave 2nd helpings of fruit and vegetables; teachers limited snacks in the classroom to low-fat and low-sugar foods, and children were encouraged to drink water instead of SSBs. Physical intervention: each child received 60 minutes of PA each day during school hours (walks outside, modifications to PE, active classroom breaks, and active recess). Parental involvement: home environment, family nights, newsletters, and motivational telephone calls	22

RCT = randomised controlled trial.

NRCT = nonrandomised controlled trial.

EN = environmental.

ED = educational.

P = physical.

PA = physical activity.

HE = healthy eating.

PI = parental involvement.

*∗* = three different studies using the same dataset.

**Table 2:** Summary of primary outcomes and moderator variables.

Location	Study ID (author et al., year, and intervention name)	Primary measures (growth reference)	Moderator variables	Primary outcomes	Moderator outcomes
Canada, British Columbia	Stock et al., 2007, Healthy Buddies [38]	BMI	X	Younger group: no change; older group: positive	X

Chile, Ñuñoa	Kain et al., 2014 [37]	BMI *z*-score (WHO)	MVPA (p)	positive	Positive

England, Wigan Borough	Fairclough et al., 2013, CHANGE! [39]	BMI, BMI *z*-score (IOTF)	PA and sedentary time (a), dietary intake (24-hour recall food intake questionnaire)	BMI, no change, BMI *z*-score, no change	Sedentary time, no change, light PA, positive, moderate PA, no change, vigorous PA, no change, and fruit and vegetable intake, no change

England, northeast	Gorely et al., 2009 GreatFun2Run [35]	BMI, BMI-SDS (UK 1990)	PA (a, p), fruit and vegetable intake (24-hour recall with interview)	BMI, positive, BMI-SDS, positive	MVPA, positive, fruit and vegetable intake, no change

England, southwest	Kipping et al., 2014, Active for Life Year 5 (AFLY5) [40]	BMI	MVPA and sedentary time (a), screen time (q), fruit and vegetable consumption (q), and high energy drink intake (q)	No change	Weekend screen time, positive, high energy drinks, positive, weekday screen time, no change, fruit and vegetable consumption, no change, MVPA, no change, and sedentary time, no change

Greece, Ioannina	Angelopoulos et al., 2009, CHILDREN Study [36]	BMI, BMI *z*-score (CDC)	dietary intake (24-hr recall with interview), PA (q)	BMI, positive, BMI *z*-score, no change	MVPA, positive, fruit intake, positive, SSBs, positive, and vegetable intake, no change

Netherlands, Rotterdam	Jansen et al., 2011, Lekker fit [42]	BMI, BMI-SDS (IOTF)	X	BMI, no change, BMI-SDS, no change	X

New Zealand, Otago	Taylor et al., 2007, APPLE [32]	BMI *z*-score (CDC)	Dietary intake (Short Food Questionnaire), PA (a), and PA and television viewing time (Physical Activity Questionnaire for Older Children)	Positive	Carbonated beverages, positive, fruit intake, positive, vegetable intake, no change, higher accelerometer counts at year 1, positive, accelerometer counts, no change, PA (q), negative, and TV viewing time, no change

New Zealand, Waikato	Rush et al., 2012, Project Energize [43]	BMI-SDS (UK 1990)	X	No change	X

Norway, southeast	Grydeland et al., 2013, Grydeland et al., 2014, and Bergh et al., 2012, HEIA [28–30]	BMI, BMI *z*-score (WHO)	PA (a)	Total group: BMI, no change, BMI *z*-score, no change; girls: BMI, positive, BMI *z*-score, positive; and boys: BMI, no change, BMI *z*-score, no change	PA, positive

Portugal	Rosário et al., 2012 [41]	BMI	Dietary intake (24-hr recall with interview), PA (q)	No change	Vegetable intake, positive, fruit intake, positive, and PA, no change

Spain, Reus, Cambrils, Salou, and Vila-seca	Tarro et al., 2014, Education in Alimentation (EdAl) [31]	BMI, BMI *z*-score (WHO)	Eating habits (self-report), after-school PA (q)	BMI, no change, BMI *z*-score, positive	Fruit and vegetable intake, no change, after-school PA in participants who engaged in >5 hours per week at baseline, positive

Spain, Granollers	Llargues et al., 2011, Avall [33]	BMI	Eating habits (FFQ and Krece Plus test), PA (q)	Positive	fruit intake, positive, vegetable intake, no change, SSBs, no change, and PA, positive

United States, Louisiana	Williamson et al., 2012, LA Health Project [34]	BMI *z*-score (CDC)	School food selection and intake (digital photography), PA, and sedentary time (Self-Administered PA Checklist)	PP total: no change; PP overweight: no change; PP + SP total: no change; PP + SP overweight: no change; EM total: no change; EM overweight: no change; and EM white girls: positive	PP: PA, no change, sedentary time, no change; PP overweight: PA, negative; PP + SP: PA, no change, sedentary time, no change; PP + SP overweight: PA, no change; and EM: PA, no change, sedentary time, no change

United States, South Dakota	Story et al., 2012, Bright Start [56]	BMI, BMI *z*-score (CDC)	X	BMI, no change, BMI *z*-score, no change	X

HB = health behaviour.

HK = health knowledge.

HA = health attitudes.

a = accelerometer.

p = pedometer.

q = questionnaire.

PA = physical activity.

MVPA = moderate vigorous physical activity.

HE = healthy eating.

SSB = sugar sweetened beverages.

WHO = World Health Organisation.

IOTF = international obesity task force.

UK 1990 = United Kingdom 1990.

CDC = center for disease control.

PP = primary prevention.

PP + SP = primary prevention + secondary prevention.

EM = environmental modification.
